# A pilot study on the effects of preventive hoof trimming on acute stress and behavioral parameters in non-lame dairy cows

**DOI:** 10.5455/javar.2026.m1058

**Published:** 2026-06-22

**Authors:** Mohammed Babatunde Sadiq, Siti Zubaidah Ramanoon, Rozaihan Mansor, Wan Mastura Mohamed Shaik Mossadeq, Sharifah Salmah Syed-Hussain

**Affiliations:** 1 Department of Farm and Exotic Animal Medicine and Surgery, Faculty of Veterinary Medicine, Universiti Putra Malaysia, 43400 UPM Serdang, Malaysia; 2 Department of Veterinary Pre-clinical Science, Faculty of Veterinary Medicine, Universiti Putra Malaysia, 43400 UPM Serdang, Malaysia; 3 Department of Veterinary Clinical Studies, Faculty of Veterinary Medicine, Universiti Putra Malaysia, 43400 UPM Serdang, Malaysia

**Keywords:** Hoof trimming, dairy cows, lameness, milk, pain, welfare

## Abstract

**Objectives:** This study investigated the effects of hoof trimming (HT) on locomotion scores, behavioral activities, cortisol levels, and milk yield in non-lame cows.

**Materials and Methods:** A total of 20 healthy Australian Friesian Sahiwal cows with normal gait and without hoof lesions were randomly allocated to 2 groups: TRIM (*n* = 10) and Control (*n* = 10). All the feet of cows in the TRIM group were trimmed using the five-step Dutch method, while the control group underwent sham trimming by restraining the limbs. Blood samples were collected before and after HT (immediately and on days 0, 1, and 2) and analyzed for serum cortisol levels. The locomotion scores, time spent lying down, feeding, and standing were also recorded using installed video cameras. Data were analyzed using descriptive statistics and repeated measures analysis of variance.

**Results:** Cortisol levels were not different (*p* > 0.05) before HT but increased significantly (*p* < 0.05) in both groups immediately after HT. TRIM recorded a significantly higher cortisol level and time spent lying down on days 1 and 2 post-HT compared to the control group. No significant difference was detected in the locomotion scores, milk yield, and time spent standing and feeding between the groups at all time points.

**Conclusions:** Preventive HT may elicit acute stress in dairy cows even in the absence of claw lesions. Ensuring cows are able to express their normal behaviors, particularly lying down, may assist in ameliorating the acute stress associated with HT.

## 1. Introduction

Lameness is a well-known cause of pain and discomfort in dairy cows, with negative impacts on behavioral activities such as lying down, feeding, and social interaction [1]. Lame cows contribute substantially to economic losses experienced by dairy farmers as a result of a decline in milk yield, low reproduction performance, and a relatively shorter production lifespan [2, 3].

Hoof lesions account for 70–90% of lameness episodes in dairy cows [4]. Although not all claw lesions elicit perturbed gait, pre-lame cows are metabolically unhealthy before the onset of lameness [5]. For instance, the concentrations of serum amyloid A, an acute phase protein, increased significantly at 8 weeks and 4 weeks before parturition in cows that later became lame postpartum [6]. Serum metabolites relating to protein metabolism were significantly altered at 4–8 weeks before calving and during the week of claw lesions and lameness diagnosis [7]. These findings reflect the need for early detection of claw lesions and effective measures to prevent their progression to clinical lameness.

Therapeutic hoof trimming (HT) is among the most commonly applied treatment modalities for hoof horn lesions [8, 9, 10]. Studies reporting the impact of therapeutic HT on the welfare of lame dairy cows suggest behavioral changes such as an increased number of steps/days, increased pressure nociceptive threshold, higher leg movements, and time allocated for lying down and feeding activities [11, 12]. Other studies reported a significant increase in levels of cortisol, haptoglobin, and fecal metabolites in lame cows compared to untrimmed groups after treatment [9, 13]. These studies suggest that HT induces varying stress levels in lame cows and the need to address the welfare issue.

Apart from therapeutic HT, preventive HT is also conducted in dairy cows to reduce the risk of lameness and ensure better hoof health [14, 15]. This practice has been associated with economic benefits and improved performance [16, 17]. Partial herd HT yielded a significant difference in 3-year net benefits of US$ 4,337 relative to the whole herd HT [17]. Dairy farms in which cows were trimmed on a yearly and biannual basis had a lower prevalence of clinical lameness compared to herds that practiced HT only on an as-needed basis [14]. Preventive HT techniques were also effective in reducing the incidence of lameness and hoof horn lesions in housed dairy cattle [15, 16, 18].

Preventive HT entails reducing the claw to its normal length, removing loose horns, and ensuring weight distribution is directed to the wall rather than the sole regions that are susceptible to hoof lesions [14]. The removal of horn tissues is performed by using electric grinders and hoof knives, thereby exposing the claw to high temperature and increased sensitivity of the horn tissue and sole region. These procedures may elicit acute stress and affect cows‘ behaviors even in the absence of claw lesions. However, evidence-based data are lacking to support this proposition, particularly in the tropics despite the practice of preventive HT in dairy herds in several tropical countries [19, 20]. In temperate and freestall dairy herds, Stoddard and Cramer [21] reported significant farm-dependent associations between HT, resting, activity behaviors, and milk yield. Numeric decreases in lying behaviors were observed on the day of HT, returning to baseline values a day post-HT. Van Hertem et al. [22] found that dairy cows spent less time interacting with herd mates on the day after HT, whereas an earlier study found signficant correlations between gait score and behavioral alterations after preventive HT [23]. Notably, these three studies were conducted in temperate countries. A study by Saikiran et al. [19] in India investigated dairy cattle behavior before and after HT, but only lame cows were recruited.

Thus, there is relatively scarce data on the stress associated with preventive HT either during or immediately after the procedure. Little is known as to whether preventive HT elicits short-term stress and behavioral changes in sound cows, particularly those without hoof lesions and reared in tropical settings. This study was designed to investigate the effects of preventive HT on locomotion scores, behavioral activities (time spent lying down, standing, and feeding), cortisol levels, and milk yield in non-lame cows.

## 2. Materials and Methods

### 2.1. Ethical approval

The protocol was reviewed and approved by the Institutional Committee for Animal Use and Care, Universiti Putra Malaysia (Ref: UPM/IACUC/AUP-R010/2019), and this manuscript was prepared based on the guidelines outlined in the REFLECT statement for reporting RCTs in livestock.

### 2.2. Study design

This study involved a randomized control trial to evaluate the short-term changes in behavioral activities, stress, and milk yield in dairy cows during and after preventive HT [24].

### 2.3. Study herd and selection criteria

The animals used in this study were selected from a dairy farm located in Serdang, Selangor, Malaysia. This farm was selected based on its large herd size (> 400 cows); proximity to the research institution; availability of on-farm lameness management facilities, electronic health records system, and farm staff trained in hoof care and lameness management. The farm manager and owner were initially briefed about the study objectives, inclusion criteria, and consent to participate. Thereafter, the farm was visited by the researchers to obtain the farm manager’s consent and ascertain if the farm conformed to the eligibility criteria. Upon certifying that the farm is eligible for the study, a consent form was signed by the farm manager.

### 2.4. Animal housing, feeding regimen, and management

The farm had a total of 522 Friesian Sahiwal and Friesian Jersey cows with an average 305-day milk yield of 5,264 L. The cows were completely housed throughout the year with a stocking density of 0.9 cow/stall. The floor at the feeding area and walkways were designed with slatted concrete floors, whereas compost was used as the bedding at the resting area. The cows were milked twice daily (i.e., 6:30 am and 5:30 pm) in a herringbone milking parlor. Floors were scraped thrice daily using an automatic scrapper running through the walkways.

Regarding lameness management, cows were trimmed for prophylaxis during early lactation (within 120 days in milk) and dry periods. The farm usually performs monthly locomotion scoring to identify newly lame cows. A formaldehyde-based solution was present at the exit of the milking parlor to disinfect cows’ feet after milking sessions. The cows were fed a total mixed ration, comprising Napier grass, soybean cake, fish meal, grain corn, and mineral supplements, depending on the stage of lactation.

### 2.5. Animal selection and enrollment criteria

The animals were selected from a barn housing 150 multiparous and primiparous lactating cows. We decided to enroll cows from a single barn for easy monitoring of behavioral activities during follow-up periods. All cows in the barn were assessed for locomotion scores after the morning milking session using the 5-point scoring system developed by Sprecher et al. [25], and those with sound locomotion (< score 3) were identified for further examination. Cows were considered for enrollment if they had a non-lame locomotion score ( < score 3), absence of hoof lesions on any of the feet, not treated for any disease or disorder two weeks before enrollment, a body condition score (BCS) of 3.0–3.5, and between 60 and 120 days in milk.

Cows that fulfilled the inclusion criteria were restrained on a hydraulic tilting table, and all feet were examined. Thereafter, by tossing a coin, cows were randomly allocated into two groups, receiving either actual HT (TRIM; *n* = 10) or sham trimming (CON; *n* = 10). Enrollment into the study was completed after two consecutive visits. Physiological parameters such as rectal temperature, respiratory rate, and rumen motility were assessed during enrollment. The cows were marked using an Estrus detector and kept in different barns for easy follow-up assessments.

### 2.6. Assessment of behavioral variables, locomotion scores, and milk yield

Behavioral measures such as lying down (min/day), standing time (min/day), feeding time (min/day), and locomotion scores of the cows were assessed at –1, 0, +1, and +2 days after HT. All the behaviors were assessed using an installed camera with the capacity to video record the cows for 72 h when fully charged. Lying-down duration was defined as the time spent by the cow in a typical lying-down position with the left or right flank touching the ground. Time spent feeding was based on sighting the cow at the feed trough while it selects, prehends, or chews food. Standing time was the duration spent in a typical standing position idly without performing other activities [10]. The five-point locomotion scoring system was applied to detect any alteration in gait, as the cows walked on a flat and non-slippery surface in the resting pen. Upon completing the study at 2 days post-HT, the video recordings were analyzed by another assessor who was unaware of the group each cow was allocated to. A sequential analysis method described by Hristov and Ivetic [26] was used in analyzing the video records. All the recorded activities were spotted by employing a five-time repeated analysis of the video records. Meanwhile, milk yield was measured in kg/L/day using the electronic milking records system on –1, 0, +1, and +2 days after HT.

### 2.7. Collection of blood samples and serum analysis

Upon restraining the animals in the HT facility, the cows were allowed to remain calm for 10 min, and the coccygeal area was swabbed with alcohol and prepared for blood sampling. A sterile 32 G needle was introduced into the coccygeal vein, 5 ml of blood was collected, and it was stored in clot activator tubes (BD Vacutainer^®^, BD, Plymouth, United Kingdom). The same volume of blood was collected after HT via the jugular vein while the cow was in lateral recumbency. Insulated containers and ice packs were used to store the blood samples, which were transported to the laboratory immediately and centrifuged at 2500 gm at 24°C for 10 min. The serum was stored at –20°C until further analyses. Single blood sampling was conducted on day 2 after HT and processed using the same approach.

The samples were thawed using a water bath at 37°C for 15 min before analysis. Serum cortisol concentration was measured using Bovine ELISA Cortisol Kits (Elabscience^®^), and the intra- and inter-assay coefficients of variation were 7.6% and 7.9%, respectively. The detectable level of cortisol ranged from 1.56 to 100 ng/ml.

The selected time periods for the blood sampling were based on previous studies reporting the changes in behavior following therapeutic trimming in lame cows [22, 23]. Extended blood sampling after day 2 post-trimming was not performed in this study, since our focus is acute stress and evidence from literature suggesting that potential behavioral alterations are short-lived within one week post-trimming.

### 2.8. Hoof trimming

The cows’ hooves were trimmed while positioned and restrained in the HT facility. First, the front legs (right before left) were restrained and trimmed before proceeding to the rear legs in the same manner. The time taken to restrain the cow and to perform the HT procedures was recorded in minutes. HT was conducted by a professional hoof trimmer trained in the application of functional HT. Upon measuring the dorsal wall length, claws were reduced to normal length (90 mm) using a hoof nipper. A grinder was then used to reduce the horn and balance the heel and sole thickness between the medial and lateral claws. The procedure was completed by pairing the typical sole ulcer site to form a dish, thus redirecting more pressure to the wall.

For the CON group, the limbs were restrained in the same order as described for the treatment group. Specifically, each of the front limbs was restrained for 120 sec, and each of the hind legs was restrained for 180 sec. Thereafter, the hooves were cleaned and disinfected using povidone-iodine. No HT was performed in the CON group.

### 2.9. Data analysis

All the statistical analyses were performed using IBM SPSS (Version 23) at the cow level. Data were first checked for normality based on the level of skewness and kurtosis. Descriptive statistics were used to summarize the data set. Variables that were normally distributed were presented in means and standard deviation, while median and interquartile range were computed for variables not conforming to normality tests. Chi-square and independent *t*-tests were applied to compare the animal-level variables between the treatment and control groups. Behavioral variables such as lying down and standing duration and time spent feeding were analyzed using repeated measures analysis of variance (ANOVA). A *p*-value of 0.05 was considered statistically significant in all the analyses.

## 3. Results

### 3.1. Study inclusions and exclusions

A total of 35 non-lame cows were selected after morning milking and assessed for enrollment. Among the examined cows, 31 met all the inclusion criteria, while the other cows had one or more hoof lesions. In total, 16 cows were trimmed (TRIM), and 15 were observed for sham trimming (CON). A total of 20 cows (10 in each group) were finally enrolled in the study, as 6 and 5 animals were excluded from TRIM and CON, respectively. The reasons for exclusion included pronounced agitation, vocalization, and leg movements during the procedure, as well as alterations in clinical parameters such as high temperature and respiratory rate either before or after the experiment.

### 3.2. Descriptive results and univariate analysis

The characteristics of the enrolled cows are summarized in Table 1. Overall, the median (IQR) LS was 1 (0), whereas the mean (± SD) time taken to restrain and treat the cows was 2.5 (± 0.2) and 9.5 (± 2.1) min, respectively. Last milk records and days in milk during enrollment were 12.8 (± 0.7) and 185.5 (± 13.3), respectively. There was no significant difference in BCS, hock condition score, locomotion score, and last recorded milk yield between the 2 groups. However, the time spent in completing the HT and the proportion of cows showing agitation were significantly higher in TRIM compared with CON. Days in milk were significantly higher in CON than in the TRIM group.

**Table 1.** Descriptive statistics of the cows’ characteristics, milk yield, and behavioral variables of the treatment and control groups at enrollment of the clinical trial (day 0).

Variables	TRIM (n = 10)	CON (n = 10)	Overall (n = 20)
Milk yield			
Last recorded milk yield/day (kg; mean ± S.E)	22.8 ± 0.9	20.9 ± 0.5	21.8 ± 0.6
DIM (mean ± SD)^a^	155.7 ± 11.7	216.0 ± 20.0	185.5 ± 13.3
BCS (Median [IQR])	3 (0)	3 (0)	3(0)
Locomotion score (Median [IQR])	1 (0)	1 (0)	1 (0)
HCS (Median [IQR])	1 (0)	1 (0)	1 (0)
Parity^a^	2 (0)	1 (0)	1 (0)
Restraint duration (min; mean ± S.E)	2.5 ± 0.2	2.4 ± 0.1	2.5 ± 0.2
Treatment duration (min; mean ± S.E)^a^	10.6 ± 0.5	8.4 ± 0.7	9.5 ± 2.1

^a^ difference between the experimental groups was significant. Notes: TRIM, trimmed cows; CON, sham-trimmed cows; SD, standard deviation; SE, standard error; IQR, interquartile range; HCS, hock condition score; BCS, body condition score; DIM, day in milk.

### 3.3. Behavioral activities and locomotion scores

Table 2 shows the mean time spent (mean ± SD) lying down, standing, and feeding during the study. All the behavioral activities (lying-down time: 10.6 vs. 10.8 h/day, standing time: 2.4 vs. 2.1 h/day, and feeding time: 3.5 vs. 3.7 h/day) were not significantly different between the two groups a day before HT. However, lying time increased significantly in the TRIM group from day 0 to +2, but no changes were observed in the CON group. The TRIM group recorded significantly higher lying time on day 0 (12.8 h/day; 95% CI 10.4–14.8 h/day), +1 day (12.6 h/day; 95% CI 10.2–14.2 h/day), and +2 days post-HT (12.5 h/day; 95% CI 10.2–14.2 h/day). Meanwhile, locomotion scores, standing time, and time spent feeding were not significantly different within and between the groups.

**Table 2.** Mean (±SD) lying-down time, time spent at the feed bunk, and standing time in actual trimmed and control cows during the study period.

Group	Day before HT	Day of HT	Day 1 post-HT	Day 2 post-HT
Time spent lying (h/day)				
TRIM	10.6 ± 2.4^a, a^	12.8 ± 3.1^b, a^	12.6 ± 3.3^b, a^	12.5 ± 2.9^b, a^
CON	10.8 ± 2.1^a, a^	10.6 ± 2.8^a, b^	10.5 ± 2.6^a, b^	10.7 ± 2.5^a, b^
Time spent feeding (h/day)				
TRIM	3.5 ± 0.6	3.3 ± 0.4	3.6 ± 0.5	3.7 ± 0.4
CON	3.7 ± 0.4	3.2 ± 0.3	3.4 ± 0.4	3.5 ± 0.5
Time spent standing (h/day)				
TRIM	2.4 ± 0.2	2.2 ± 0.4	2.0 ± 0.4	2.1 ± 0.5
CON	2.1 ± 0.3	2.3 ± 0.3	2.1 ± 0.2	2.3 ± 0.4
Locomotion scores (median scores)				
TRIM	1 ± 0	1 ± 0	1 ± 0	1 ± 0
CON	1 ± 0	1 ± 0	1 ± 0	1 ± 0

**Note:** Values are presented as mean and standard deviation. Comparisons are within and between groups, denoted by the first and second superscripts, respectively. Superscripts with different letters are statistically significant.

### 3.4. Serum cortisol levels and milk yield

There was no significant difference in the serum cortisol levels (ng/ml) between the groups on day 0 and 5 min before HT (Table 3). In both groups, serum cortisol increased significantly immediately after HT while the cows were still restrained in a lateral position. Meanwhile, the increased cortisol levels in TRIM were maintained until day 2 post-HT, while those of CON decreased significantly to normal levels on days 1 and 2. Comparisons between both groups revealed that cortisol levels were significantly higher in TRIM on days 1 and 2 post-HT than in the CON group. Table 3 also depicts the average daily milk yield during the study period. The CON group produced 2.0 kg and 2.2 kg more milk/day on days 1 and 2, respectively, than the TRIM, but the difference was not statistically significant (*p* < 0.05). Overall, no significant difference was observed in the milk yield between the groups.

**Table 3.** Mean (± SE) serum cortisol levels in TRIM and CON groups before and after HT.

Group	Cortisol (ng/ml)				
	Day before HT	5 min before HT	After HT	+1 day post-HT	+2 days post-HT
TRIM	32.4 ± 3.6^a, a^	34.9 ± 3.9^a, a^	80.5 ± 3.0^b, a^	62.2 ± 3.1^b, a^	56.5 ± 2.3^b, a^
CON	27.0 ± 3.1^a, a^	28.0 ± 3.4^a, a^	77.5 ± 4.2^b, a^	32.8 ± 2.8^a, b^	35.1 ± 2.6^a, b^
Milk yield (kg/day)					
Groups	Day before HT	Day 0	Day 1	Day 2	
TRIM	21.9 ± 0.7^a, a^	20.5 ± 0.5^a, a^	21.4 ± 0.5^a, a^	21.8 ± 0.6^a, a^	
CON	23.4 ± 0.6^a, a^	22.5 ± 0.9^a, a^	22.6 ± 0.9^a, a^	22.5 ± 0.9^a, a^	

**Note:** Values are presented as mean and standard deviation. Comparisons are within and between groups, denoted by the first and second superscripts, respectively. Superscripts with different letters are statistically significant.

## 4. Discussion

This study revealed the acute transient stress experienced by a cow after preventive HT. Based on the findings, there is a considerable effect of HT on the stress response, as evident by the changes in cortisol levels. Accordingly, serum cortisol was not significantly different a day before and 5 min before HT between the TRIM and CON groups but increased significantly in both groups after HT, and the increment was maintained in TRIM until day 2 post-HT. These findings indicate that both restraining, as in CON, and horn removal during trimming led to acute stress, but the effect was more evident during the latter process.

Previous studies have reported increased concentrations of serum and plasma cortisol in dairy cows after HT compared to baseline [27, 28]. In a study conducted by Rizk et al. [27], plasma cortisol returned to baseline between 3.00 and 3.25 h after HT. Nevertheless, cortisol levels were only monitored at approximately 24 h intervals in the present study.

Our results are slightly different compared to other studies where cortisol concentrations were observed following more painful procedures like dehorning and lameness induction [28, 29]. In both experiments, peak cortisol concentrations (35 ng/ml in calves and 63.5 ng/ml in cows) were achieved after dehorning and lameness induction, and the levels were maintained 48 h after dehorning. Dehorning and lameness induction are associated with pain and inflammation, which translates to different stress levels compared to preventive HT.

Among the behavioral activities assessed in this study, only lying down time differed significantly between the two groups after HT. Specifically, the TRIM group spent more time lying after HT on days 1 and 2 after HT compared to the CON group, whereas lying time was not affected in the CON group. These findings align with previous studies in which non-lame dairy cows spent more time lying immediately after HT and up to a few weeks later [30, 31, 32] and reduced activity on the day after HT [17]. However, the time taken for cows to return to their normal lying duration may differ between studies due to bedding type, HT method applied, and cow-level factors [29, 33]. The additional lying time (2 h/day) recorded in the TRIM cows differs slightly compared to the values reported in previous studies [34, 35], which might be explained by variation in hoof health, housing design, and cow-level factors such as stage of lactation, parity, and BCS. Nevertheless, neither parity nor stage of lactation varied significantly between the TRIM and CON in this study.

The longer lying duration on days 1 and 2 post-HT in the TRIM group reflects the stress-related effects of HT and decreased comfort. Such changes are probably more due to adjustment to a new pattern angle and sole thickness upon completing the HT rather than the handling procedure, since both groups were restrained in the same manner. Changes in weight distribution and gait properties that are not detectable using subjective methods may also contribute to the present finding. Previous studies demonstrated that increased lying time is associated with higher locomotion scores [35], and the latter correlates with increased weight-shifting behavior—an indication of pain [31]. A recent study also revealed that block application led to significant changes in weight distribution across the limbs in both non-lame and lame cows [36]. In contrast, Cutler et al. [2] found that therapeutic blocks had significant effects on locomotion scores but no evidence to suggest any impact on lying items. Changes in weight distribution arising from preventive HT and block application and their association with lying activities need to be elucidated.

Locomotion scores and time spent feeding were not different between the groups during the follow-up period. This outcome agrees with the previous findings in which locomotion scores and feeding time remained the same after HT in non-lame cows [30, 31, 35]. These results are not surprising given the plethora of studies on the relationship between higher locomotion scores (lame cows) and feeding or ruminating time. Alterations in feeding and rumination behavior in lame cows stem from lower visits to the feed bunk and metabolic and inflammatory changes, particularly during the transition method [1]. These events are not expected in the TRIM group since the cows were clinically healthy, had no hoof lesions, and were only subjected to preventive HT.

Similarly, milk yield was not affected by restraint or actual HT based on the comparison between the basal and 2-day observations in the TRIM and CON groups. The lack of effects is unsurprising; several factors influence milk yield in dairy cows, and a large effect size is necessary for any single factor to elicit a measurable change. Likewise, a recent study found no significant effect of preventive HT on milk yield in dairy cows [31]. In contrast, cows with hoof lesions recorded a significant decline in milk yield days after therapeutic HT [16], which might stem from the higher stress response during horn removal.

The limitations of this study include the small sample size, short-term assessment of stress hormones, and several other behavioral activities that were not considered. Although the animals were randomly allocated to TRIM and CON, the relatively small sample size of 10 cows in each group may reduce the chances of obtaining significant results for some of the variables. Some cows were excluded from the study during the recruitment stage due to pronounced agitation, vocalization, and excessive leg movements. While the removal of these cows may introduce selection bias, the decision is supported by previous research reporting the effects of such disturbances on stress-related hormones and biomarkers, thereby introducing noise to the actual measured variables [22, 23]. Removing the cows with pronounced agitation increased the odds of attributing the behavioral and stress-related alterations to the preventive HT rather than animal handling.

Furthermore, the study duration was from one day before to two days post-HT, indicating that the long-term effects of HT cannot be elucidated from the findings. Behavioral activities such as neck activity, rumination, and grooming are other vital behaviors that could be considered in future studies. The HT procedure employed in this study was the 5-step Dutch method [14]; findings may differ following the application of other HT methods. This is because the extent of pairing when reducing the horn tissues and dorsal wall length varies between HT techniques, which could influence the weight distribution between the medial and lateral claws [15].

## 5. Conclusions

In conclusion, this study revealed that both cows’ restraint and the HT procedure elicited acute stress in non-lame dairy cows, but the stress response was more pronounced following the latter procedure. This was evident in one of the behavioral parameters, increased lying time, as well as higher levels of serum cortisol in the HT group. The present findings suggest that HT elicits acute stress in dairy cows even in the absence of hoof lesions. Farmers and veterinarians may consider providing comfortable lying-down areas to assist cows in ameliorating the associated stress after preventive HT. Nevertheless, given the limitations of this study, a larger-based, multicenter trial is recommended to validate the findings. Reliable and diverse markers of pain and stress may be assessed in future studies. Lame cows and those affected with hoof lesions can also be included in the treatment groups for more comprehensive comparisons and results. Addressing these limitations will assist in generalizing the findings to tropical dairy farms.

## Data Availability

The data presented in this study are available from the corresponding author upon reasonable request.

## List of abbreviations

HT, hoof trimming; TRIM, trimmed group; CON, control group; CI, confidence interval; SD, standard deviation; SE, standard error; IQR, interquartile range; HCS, hock condition score; BCS, body condition score; LS, locomotion score; DIM, day in milk; REFLECT, Reporting Guidelines for Randomized Control Trials; h, hours; min, minutes; sec, seconds; gm, grams; ng/ml, nanograms per milliliter; h/day, hours per day; kg/day, kilograms per day.
